# A Study of Teacher Stereotypes: How Do Tuition-Free Teacher Candidates and General Undergraduates Think about Middle School and University Teachers in China?

**DOI:** 10.3389/fpsyg.2017.00576

**Published:** 2017-04-19

**Authors:** Youxia Zuo, Yufang Zhao, Chunhua Peng, Youguo Chen

**Affiliations:** ^1^Center of Studies for Psychology and Social Development, Key Laboratory of Cognition and Personality (Ministry of Education), Faculty of Psychology, Southwest UniversityChongqing, China; ^2^Center for Studies of Education and Psychology of Minorities in Southwest China, Southwest UniversityChongqing, China; ^3^Laboratory of Cognition and Mental Health, Chongqing University of Arts and SciencesChongqing, China

**Keywords:** tuition-free teacher candidates, stereotypes, occupational cognition, occupational personality, occupational emotions, middle school teacher

## Abstract

A tuition-free teacher candidate is an undergraduate who receives tuition-free teacher education and must work as a teacher in a middle school after their graduation. Tuition-free candidates are of the focus of many researchers; however, no study reports how tuition-free teacher candidates think about teachers. The present study explored stereotypes about middle school and university teachers held by teacher candidates. Specifically, we looked for the differences between the stereotypes held by the teacher candidates and general undergraduates. This study attempted to provide a potential tool to predict the actual willingness of teacher candidates to work as middle school teachers. University and middle school teachers were evaluated using descriptive phrases or words on a five-point Likert scale by 116 tuition-free teacher candidates and 155 general undergraduates. Exploratory factor analyses revealed a three-factor stereotype model including occupational cognition, occupational personality, and occupational emotion. Compared with general undergraduates, teacher candidates held more positive occupational personality and emotions toward middle school teachers; they held more negative occupational emotions toward university teachers. Further, the undergraduates' willingness to be middle school teachers positively correlated with positive occupational emotions and negatively correlated with negative occupational personality and emotions toward middle school teachers. This supported previous studies that individuals' professional willingness were influenced by their stereotypes about professions.

## Introduction

In 2007, the Chinese Ministry of Education implemented a policy of tuition-free teacher education to improve the quality of basic education. Many undergraduates in teacher-training college do not pay tuition and lodging fees and receive cost of living bonuses supported by state revenue. However, often, these undergraduates cannot afford to make career choices, and return to their hometown and work in local secondary schools as middle school teachers for more than 10 years after their graduation. They are known as tuition-free teacher candidates. Currently, there are approximately 90,000 such candidates (Weng and Qi, 2014). However, many tuition-free teacher candidates do not like teaching (Zhang and Zheng, 2007; Wu et al., 2010). Some of them plan to reimburse the tuition in the future so that they can enjoy the freedom of occupation selection; others have no clear plan about their future work (Lian, 2007; Wang, 2010). Obviously, teacher candidates will perform better in their future careers if they want to be a teacher. Thus, it is important to know whether teacher candidates want to be middle school teachers. However, some students hide their real thoughts about preferred professions and report that they want to be teachers because of economic reasons and parents' orders (Zhang and Zheng, 2007). We attempted to identify the stereotypes about middle school teachers held by teacher candidates and then used them to predict whether they wanted to be middle school teachers. Furthermore, it may help to select appropriate teacher candidates who ardently want to be a teacher.

People usually use stereotypes to simplify cognitions about individuals or certain ways of doing things. A stereotype is a cognitive structure that includes a set of beliefs about personal attributes of people in a social group. These personal attributes include personality traits, feelings, physical appearances, preferences, and occupations (Ashmore and Boca, 1979; Wong, 2004; Kanahara, 2006). The contents of stereotypes have been widely investigated (Anderson and Sedikides, 1991; Edwin and Hub, 1999). The stereotype content model (SCM) proposed that stereotypes are based on two dimensions: warmth and competence. One may view a group as warm but not competent (e.g., the old are nice but weak) or as competent but not warm (e.g., the rich are cold but efficient) (Fiske et al., 2002). This model has been supported by some studies. For example, the SCM of immigrant subgroups predicts that perception centers on competence and warmth and relates to targets' perceived status and competition within society (Lee and Fiske, 2006). In addition, warmth and competence can reliably differentiate social group stereotypes in individualist and collectivist nations (Cuddy et al., 2009). However, some researchers still doubt whether the contents or structures of stereotypes are limited to warmth and competence (Zuo et al., 2006). Leach et al. (2007) suggested that the warmth dimension encompasses two distinct aspects called sociability and morality; they concluded that morality is more important than sociability and competence traits when giving positive in-group evaluations. It also should be noted that some researchers have tended to use the label “morality” in place of “warmth” (Vonk, 1996; Wojciszke, 2005). In addition, other components can also be found for stereotypes of Chinese people. For example, one study extracted 10 dimensions for impressions of Chinese people: morality, wealth, attitude, reason-politeness, friendship ties, skin color, temperament, sense of community, appearance, and male chauvinism (Sun, 2004). That is to say, the SCM (warmth and competence) may include necessary components of various stereotypes, rather than representing all stereotypes.

Consistent with the SCM model, warmth and competence are two necessary dimensions for teacher stereotypes. Teachers are stereotyped as being incompetent and warm (Carlsson and Björklund, 2010). Specifically, they are presented as being poorly adjusted (Erskine and Andrew, 1951) and incapable of anything but teaching (Lieberman, 1956). However, teachers are also evaluated as being warm, because they are amicable and have a deep love for students (Yuan et al., 2005). Additionally, some other features considered to befit teachers include being responsible, having profound knowledge, disseminating traditional culture or existing knowledge, and fostering social, economic, and cultural development (Lin, 1994; Yuan et al., 2005). However, teachers usually experience some degree of poverty and they often solve this problem by leaving the profession (Gerbner, 1963). The occupation of teaching is seen as having a feminine orientation; thus the male teacher is considered as low in status, poor in terms of public affairs, and as lacking assertiveness, firmness, strength, activity, and confidence by college students (O'Dowd and Beardslee, 1961). Consequently, we inferred that the SCM (warmth and competence) contained necessary components of teacher stereotypes, and the stereotypes may also include other specific attributes of teachers.

Occupational stereotypes can not only provide quick and easy assumptions that will affect the behavior of social group members (Yzerbyt and Demoulin, 2010), but also have been shown to influence individuals' career development and aspirations (Sue and Kirk, 1972; Leong, 1985). Consequently, it is valuable to identify stereotypes about teachers held by teacher candidates. We can infer that teacher candidates' stereotypes about teachers will have an important influence on their desire for work and professional behavior.

In the present study, we focused on teacher candidates' stereotypes toward Chinese middle school teachers. To better understand these stereotypes, we compared stereotypes about university and middle school teachers. In addition, previous studies suggested that positive and negative descriptions could simultaneously exist in stereotypes about a social group (Greenwald et al., 2002). In order to enhance the credibility of the present study, we explored whether the same structure would be found in positive and negative descriptions. Furthermore, a study reported that the stereotype of accountants held by accountants was significantly different from people in other occupations (Imada et al., 1980); thus, it is hypothesized that the stereotype about teachers held by tuition-free teacher candidates is different from general undergraduates. Finally, we computed correlation coefficients between undergraduates' willingness to be middle school teachers and their stereotypes about middle school teachers and predicted that students with positive middle school teacher stereotypes would be more likely to be middle school teachers.

## Materials and methods

### Participants

Twenty tuition-free teacher candidates and 20 general undergraduates participated in a free**-**association questionnaire survey. Then, 130 candidates (72 men and 58 women) and 161 general undergraduates (40 men and 121 women) participated in the formal questionnaire survey. Participants were recruited from Southwest University and ranged in age from 18 to 23 years (116 candidates and 155 general undergraduates had no missing data and were included in the final analyses). The Ethics Committee of Southwest University approved the study. Written informed consent was obtained from each participant, who received remuneration for participating in this survey.

### Procedure and measures

Free association and Likert scales were adopted to measure the stereotypes. They have been widely used as direct measurement methods of stereotypes in previous studies (Niemann et al., 1994; Angermeyer and Matschinger, 2005).

Forty participants were asked to write freely associated descriptive words or phrases about middle school and university teachers (see Supplementary Materials). Then, each word or phrase was assessed consistent with a combination of reported frequency and the suggestions of specialists and scholars in social and personality psychology. Finally, 32 positive and 32 negative words or phrases were retained for the formal questionnaire (see Appendix).

Middle school and university teachers were separately evaluated with descriptive items. Participants (*N* = 271) responded to items using a five-point Likert scale (1 = very uncharacteristic; 5 = very characteristic). At the same time, students' willingness to be middle school teachers were ranked using a five-point Likert scale (1 = unlike very much; 5 = like very much). The order of the evaluation of the two teacher types was counterbalanced between participants with an ABBA design.

### Data analysis

After data reduction finished, we used SPSS 16.0 (SPSS Inc.) for exploratory factor analysis to explore the latent psychological structure of stereotypes about teachers held by undergraduates. Because each participant (teacher candidates or general undergraduates) was asked to evaluate three occupational factors of two types of teachers, respectively, a mixed ANOVA was conducted on the positive and negative descriptions, respectively, to better understand the stereotypes about teachers. The ANOVA factors were groups (tuition-free teacher candidates and general undergraduates), teachers (middle school and university teachers), and occupational factors (cognition, personality, and emotion). The teacher and occupational factors were within-subjects factors, and the group was a between-subjects factor. The Greenhouse–Geisser correction was used to correct for any violations of sphericity (Greenhouse and Geisser, 1959), and the partial eta squared (η_*p*_^2^) was used to estimate the ANOVA effect size (Levine and Hullett, 2002).

## Results

### Exploratory factor analysis

Exploratory factor analysis was conducted in the following four situations, respectively: positive and negative descriptions of university teachers and positive and negative descriptions of middle school teachers. Principal component analysis was employed to extract the factors. Based on eigenvalues (>1), scree plot tests, the total variance explained ratio, and factor interpretability (Williams et al., 2010), we selected a three-factor model. Subsequently, we used varimax orthogonal rotation and selected items and factors based on the following criteria: (1) one item should not load on two or more factors; (2) one item's loading on a factor should be more than 0.4; (3) each factor should have at least four items; and (4) items significantly different from other items should be avoided (Gorsuch and Hao, 1993).

The exploratory factor analysis was conducted on positive and negative description scores for middle school teachers. For positive descriptions of middle school teachers (Table 1), the Kaiser-Meyer-Olkin (KMO) measure of sampling adequacy was 0.842. Three factors explained 42.506% of the total variance. The first factor reflected personality characteristics of middle school teachers, such as enthusiastic, friendly, and confident, so it was named “occupational personality” (*n* = 11, eigenvalue = 5.615, 23.396% of variance explained). The second factor reflected middle school teachers' emotional states toward their jobs, such as leisurely, loose, and less anxious, so it was named “occupational emotion” (*n* = 7, eigenvalue = 2.985, 12.438% of variance explained). The third factor reflected cognitions about middle school teacher positions, such as well-paid, prestigious, and better benefits, so it was named “occupational cognition” (*n* = 4, eigenvalue = 1.601, 6.671% of variance explained).

**Table 1:** **Exploratory factor analysis of positive descriptions of middle school teachers**.

**Items**	**Factor 1**	**Factor 2**	**Factor 3**
Enthusiastic	0.741		
Friendly	0.707		
Enterprising	0.679		
Just	0.639		
Democratic	0.627		
Confident	0.625		
Erudite	0.555		
Facetious	0.539		
Outstanding	0.504		
Unobtrusive	0.488		
Wide social	0.472		
Effortless		0.673	
Leisurely		0.661	
Worry free		0.614	
Less anxious		0.597	
Loose		0.570	
Comfortable		0.561	
Relaxed		0.544	
Well pay			0.690
Prestigious			0.586
Promising			0.557
Better benefits			0.518

For negative descriptions (Table 2), the KMO measure of sampling adequacy was 0.822. Three factors explained 42.280% of the total variance. The first factor, named occupational personality, reflected the personality characteristics of middle school teachers, such as arrogant, outdated, and indifferent (*n* = 12, eigenvalue = 4.870, 21.175% of variance explained). The second factor, named occupational emotion, reflected middle school teachers' emotional states toward their jobs, such as worried, busy, and laborious (*n* = 6, eigenvalue = 3.423, 14.881% of variance explained). The third factor, named occupational cognition, reflected individual cognitions about the middle school teacher profession, such as poor pay and inadequate benefits (*n* = 4, eigenvalue = 1.432, 6.224% of variance explained).

**Table 2:** **Exploratory factor analysis of negative descriptions of middle school teachers**.

**Items**	**Factor 1**	**Factor 2**	**Factor 3**
Arrogant	0.656		
Autocratic	0.624		
Outdated	0.623		
Greedy	0.605		
Mediocre intelligence	0.604		
Inferior	0.588		
Indifferent	0.571		
Severe	0.543		
Partial	0.520		
Luxurious	0.507		
Conservative	0.449		
Narrow social	0.435		
Worried		0.742	
Arduous		0.672	
Laborious		0.660	
Stressful teaching		0.648	
Busy		0.625	
Apprehensive		0.552	
Poor pay			0.659
Poor working environment			0.542
Inadequate benefits			−0.513
Irregular routine			0.410

Exploratory factor analysis revealed that the latent structure of stereotypes of university teachers was the same as middle school teachers. For positive descriptions of university teachers (Table 3), the KMO measure of sampling adequacy was 0.876. Three factors explained 45.401% of the total variance. The first factor was occupational cognition (*n* = 10, eigenvalue = 6.747, 29.337% of variance explained). The second factor was occupational personality (*n* = 7, eigenvalue = 1.953, 8.491% of variance explained). The third factor was occupational emotion (*n* = 6, eigenvalue = 1.742, 7.573% of variance explained).

**Table 3:** **Exploratory factor analysis of positive description of university teacher**.

**Items**	**Factor 1**	**Factor 2**	**Factor 3**
Better benefits	0.741		
Promising	0.688		
Good working environment	0.686		
Well pay	0.619		
Open	0.595		
Free	0.590		
High achievement	0.560		
Prestigious	0.549		
Elegant	0.526		
Secure	0.513		
Frugal		0.666	
Just		0.646	
Friendly		0.595	
Enthusiastic		0.589	
Unobtrusive		0.579	
Outstanding		0.537	
Democratic		0.484	
Less anxious			0.677
Leisurely			0.667
Labor-saving			0.638
Worry free			0.601
Relaxed			0.591
Loose			0.553

For negative descriptions of university teachers (Table 4), the KMO measure of sampling adequacy was 0.837. Three factors explained 43.479% of the total variance. The first factor was occupational cognition (*n* = 8, eigenvalue = 5.945, 25.848% of variance explained). The second factor was occupational personality (*n* = 6, eigenvalue = 2.344, 10.190% of variance explained). The third factor was occupational emotion (*n* = 8, eigenvalue = 1.711, 7.441% of variance explained).

**Table 4:** **Exploratory factor analysis of negative description of university teacher**.

**Items**	**Factor 1**	**Factor 2**	**Factor 3**
Low fame	0.701		
Vulgar	0.678		
Restricted	0.670		
Poor pay	0.668		
Low achievement	0.660		
Inadequate benefits	0.588		
Narrow prospect	0.534		
Poor working environment	0.513		
Luxurious		0.754	
Arrogant		0.643	
Greedy		0.613	
Autocratic		0.595	
Partial		0.521	
Indifferent		0.505	
Busy			0.757
Laborious			0.600
Stressful teaching			0.585
Worried			0.578
Apprehensive			0.530
Tough			0.516
Rigorous			0.477
Stressful researching			0.417

### Reliability and validity of questionnaire

Cronbach's alpha coefficients of the questionnaire were calculated for the four parts: positive and negative descriptions of university teachers and positive and negative descriptions of middle school teachers (Table 5). Coefficients were over 0.8 for each part and over 0.6 for each dimension in every part; this indicates satisfactory internal consistency reliability (Wright et al., 2004).

**Table 5:** **Cronbach's alpha of questionnaire of teachers' occupational stereotypes**.

**Factors**	**Occupational cognition α**	**Occupational personality α**	**Occupational emotion α**	**Total α**
Positive descriptions of university teachers	0.852	0.733	0.706	0.873
Negative descriptions of university teachers	0.832	0.756	0.723	0.852
Positive descriptions of middle school teachers	0.665	0.848	0.774	0.854
Negative descriptions of middle school teachers	0.606	0.819	0.804	0.825

The correlations between each dimension and the total questionnaire ranged from 0.3 to 0.8 and the correlations between each dimension ranged from 0.1 to 0.6; this indicates that the questionnaire structure was appropriate (Tucker, 1946). We analyzed structure validity by means of correlation matrices (Cronbach and Meehl, 1955). As shown in Tables 6, 7, the correlation coefficients between each dimension were from 0.1 to 0.6 and the correlation coefficient between each dimension and the whole dimension were from 0.65 to 0.82. These results indicated that the structural validity of questionnaire was favorable.

**Table 6:** **Correlation coefficient matrix of positive descriptions**.

	**Factor**	**Occupational cognition**	**Occupational personality**	**Occupational emotion**
University teachers	Occupational cognition			
	Occupational personality	0.539^**^		
	Occupational emotion	0.405^**^	0.290^**^	
	Average of positive descriptions	0.819^**^	0.772^**^	0.746^**^
Middle school teachers	Occupational cognition			
	Occupational personality	0.377^**^		
	Occupational emotion	0.387^**^	0.314^**^	
	Average of positive descriptions	0.755^**^	0.721^**^	0.790^**^

** *p < 0.01*.

**Table 7:** **Correlation coefficient matrix of negative descriptions**.

	**Factor**	**Occupational Cognition**	**Occupational personality**	**Occupational emotion**
University Teachers	Occupational cognition			
	Occupational personality	0.524^**^		
	Occupational emotion	0.288^**^	0.299^**^	
	Average of negative descriptions	0.799^**^	0.801^**^	0.685^**^
Middle School Teachers	Occupational cognition			
	Occupational personality	0.432^**^		
	Occupational emotion	0.201^**^	0.136^**^	
	Average of negative descriptions	0.777^**^	0.722^**^	0.631^**^

** *p < 0.01*.

### Comparisons of stereotypes between two types of college students

**Figure 1:**
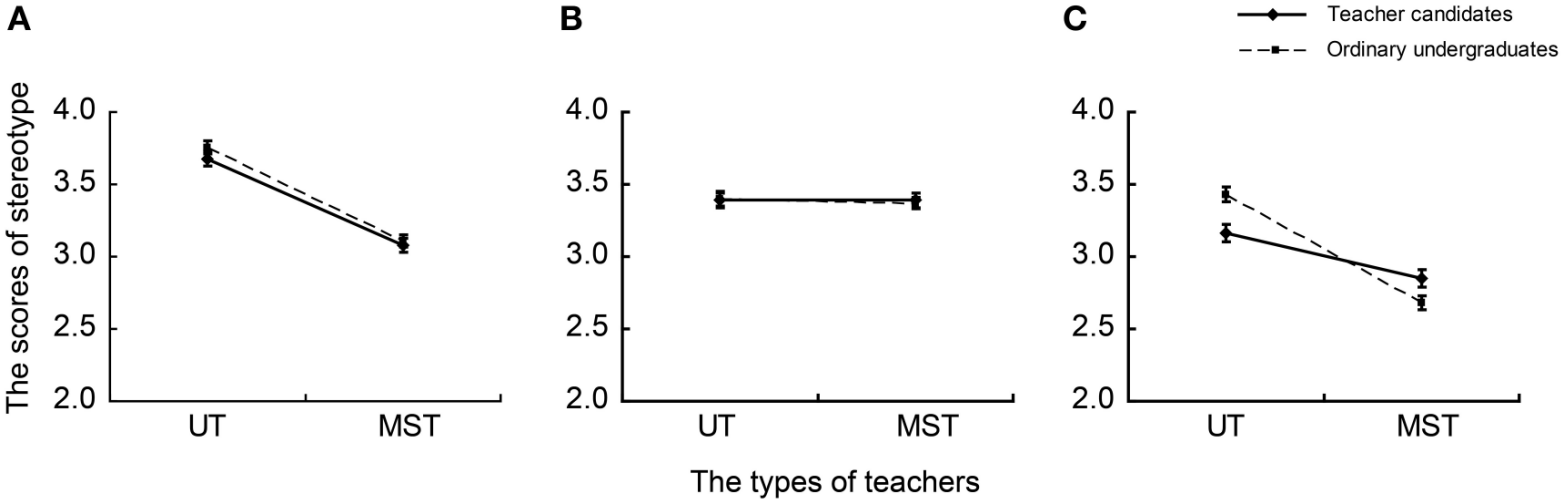
**Average scores of teachers' occupational stereotypes with positive description held by two student types**. UT, university teacher; MST, middle school teacher. **(A)** Occupational cognition; **(B)** occupational personality; **(C)** occupational emotion.

An ANOVA on the positive description score stereotypes revealed not only significant main effects of teachers [*F*_(1, 269)_ = 161.604, *p* < 0.001, η_*p*_^2^ = 0.375] and occupational factors [*F*_(2, 538)_ = 109.626, *p* < 0.001, η_*p*_^2^ = 0.290] but also significant interactions of groups × teachers [*F*_(1, 269)_ = 8.072, *p* < 0.01, η_*p*_^2^ = 0.029], teachers × occupational factors [*F*_(2, 538)_ = 78.053, *p* < 0.001, η_*p*_^2^ = 0.225], and groups × teachers × occupational factors [*F*_(2, 538)_ = 9.829, *p* < 0.001, η_*p*_^2^ = 0.035]. Simple effect tests were conducted on the groups × teachers × occupational factors interaction. In terms of occupational emotion, the scores given by teacher candidates were significantly higher than those given by general undergraduates when evaluating middle school teachers [*F*_(1, 269)_ = 4.886, *p* < 0.05, η_*p*_^2^ = 0.018]; scores given by teacher candidates were significantly lower than those given by general undergraduates when evaluating university teachers [*F*_(1, 269)_ = 12.855, *p* < 0.001, η_*p*_^2^ = 0.046] (Figure 1C). Furthermore, for occupational cognition (Figure 1A) and personality (Figure 1B), there were no significant differences between the two student types when evaluating middle school and university teachers.

**Figure 2:**
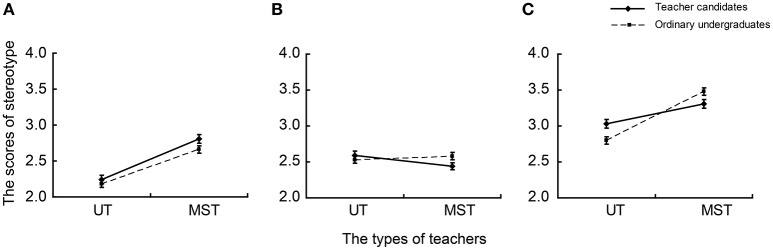
**Average scores of teachers' occupational stereotypes with negative description held by two student types**. UT-university teacher, MST-middle school teacher. **(A)** Occupational cognition; **(B)** occupational personality; **(C)** occupational emotion.

For negative stereotype descriptions, an ANOVA revealed significant main effects of teachers [*F*_(1, 269)_ = 119.032, *p* < 0.001, η_*p*_^2^ = 0.307] and occupational factors [*F*_(2, 538)_ = 222.287, *p* < 0.001, η_*p*_^2^ = 0.452] and significant interactions of groups × teachers [*F*_(1, 269)_ = 8.786, *p* < 0.01, η_*p*_^2^ = 0.032], teachers × occupational factors [*F*_(2, 538)_ = 78.655, *p* < 0.001, η_*p*_^2^ = 0.226], and groups × teachers × occupational factors [*F*_(2, 538)_ = 11.576, *p* < 0.001, η_*p*_^2^ = 0.041]. Simple effect tests were conducted on the groups × teachers × occupational factors interaction. In terms of occupational emotion, the scores given by teacher candidates were significantly lower than those given by general undergraduates when evaluating middle school teachers [*F*_(1, 269)_ = 5.381, *p* < 0.05, η_*p*_^2^ = 0.020]; when evaluating university teachers, the scores given by teacher candidates were significantly higher than those given by general undergraduates [*F*_(1, 269)_ = 9.617, *p* < 0.01, η_*p*_^2^ = 0.035] (Figure 2C). As for occupational personality, teacher candidates gave significantly lower scores for middle school teachers than general undergraduates [*F*_(1, 269)_ = 4.326, *p* < 0.05, η_*p*_^2^ = 0.016], but they did not give significantly discrepant scores for university teachers compared with general undergraduates (*p* > 0.05; Figure 2B). In addition, for occupational cognition (Figure 2A), there were no significant differences between the two student types when evaluating middle school and university teachers.

### Correlations of stereotypes and professional desire

We computed Spearman rank correlation coefficients between positive (or negative) stereotype descriptions toward middle school teachers and one's willingness to be a middle school teacher. For positive stereotypes, undergraduates' willingness to be middle school teachers positively correlated with occupational emotion (*r* = 0.148, *p* < 0.05). For negative stereotypes, undergraduates' willingness to be middle school teachers negatively correlated with negative occupational personality and emotions (*r* = −0.177, *p* < 0.05; *r* = −0.142, *p* < 0.05).

## Discussion

The present study explored stereotypes about middle school and university teachers, comparing them between regular undergraduates and tuition-free teacher candidates. With both positive and negative descriptions, exploratory factor analysis revealed a consistent three-factor model (occupational cognition, personality, and emotion) for stereotypes of two teacher types (middle school and university). Our data also revealed some differences between teacher candidates and general undergraduates in the specific stereotypes of two teacher types. Students' stereotypes of middle school teachers have a significant correlation with their willingness to be middle school teachers.

The SCM focuses on two dimensions: competence and warmth (Fiske et al., 2002). The essence of competence is confidence, independence, competitiveness, and intelligence. The essence of warmth is tolerance, good-naturedness, and sincerity. This two dimensional hypothesis has already been verified by static assessment, dynamic change (Cuddy et al., 2004; Lin et al., 2005), and cross-cultural evidence (Fiske et al., 2002). In fact, competence and warmth can be summarized by occupational personality in the present study. Specifically, the words “enthusiastic,” “friendly,” and “facetious” and so on were positively related to warmth; the words “arrogant,” “autocratic,” and “severe” and so on were negatively related to warmth. The words “enterprising,” “erudite,” and “confident” and so on were positively related to competence; the words “mediocre intelligence,” “inferior,” and “conservative” and so on were negatively related to competence. Thus, the present study provided evidence that warmth and competence exist in stereotypes held by Chinese people.

Stereotypes were not limited to a set of beliefs about the personal attributes of a group, but also included feelings, physical appearances, preferences, and occupations (Ashmore and Boca, 1979; Wong, 2004). In addition to occupational personality according to classical SCM, the present study also revealed two factors named occupational cognition and emotion in teachers' stereotype content. Occupational cognition referred to one's cognition about a specific occupation, such as welfare, remuneration, social prestige, development prospects, etc. As for occupational cognition, the evaluations of the two teacher types all involved salary, welfare, prospects, fame, and working atmosphere. However, the evaluations of university teachers considered more than the foregoing, such as freedom (corresponding words are “free,” “restricted,” and “open”), achievement (corresponding phrases are high and low achievement), and working artistry (corresponding words are “elegant” and “vulgar”). The results are consistent with a previous study that found occupational prospects, working strength, working conditions, and incomes have important effects on occupational cognition (Chen, 2011). In this paper, occupational emotion referred to cognitions about teacher's emotional states when they were working. For example, undergraduates think that middle school teachers are always anxious; this is undergraduates' cognition about teachers' emotions. The occupational emotions of two teacher types included labor intensity (corresponding words or phrases are “effortless,” “arduous,” “leisurely,” “busy,” “relaxed,” “laborious,” “stressful teaching,” and “stressful researching”) and teachers' personal emotions (corresponding words or phrases are “worry-free,” “worried,” “less anxious,” and “apprehensive”). Adelmann (1989) suggested that higher emotion workers belonged to the teaching profession and middle school teachers suffered from students' bad behavior, time pressure, excessive workload, and so on. The present study suggests that occupational cognition and emotion are parts of teacher stereotypes held by undergraduates.

For both the positive and negative descriptions of occupational cognition, there were no significant differences between teacher candidates and general undergraduates when evaluating middle school and university teachers. Generally, people's beliefs about others partly depend on their experience. Stereotypes also result from the complicated interaction between their holder and targets (Hakel et al., 1970; Imada and London, 1979). To some degree, the two student types have contact with the same type of middle school and university teachers. Thus, they may have received similar information about teachers' salaries, welfare, prospects, and so on. In addition, there were mutual contacts and sharing of viewpoints between teacher candidates and general undergraduates; thus, it is natural that they have similar occupational cognitions toward the two teacher types.

In terms of the occupational personality of middle school teachers, teacher candidates gave significantly lower scores than undergraduates in the context of negative descriptions (Figure 2B). Similarly, for occupational emotion about middle school teachers, compared with general undergraduates, teacher candidates had significantly higher scores in the context of positive descriptions (Figure 1C) and significantly lower scores in the context of negative descriptions (Figure 2C). The results are consistent with social identity theory (Tajfel, 1981, 1982) that social identity consists of social categorization, social comparison, and positive distinctiveness (Tajfel, 1982). In emerging intergroup comparisons, individuals would increase self-esteem by preserving or achieving positive social identity. Simultaneously, individuals tended to give in-group members more positive appraisal. In addition, even with no explicit conflict and competition in groups, there is also a tendency toward in group-favoring behavior (Tajfel, 1974, 1981; Tajfel and Turner, 1979). Therefore, compared with general undergraduates, teacher candidates held a superior stereotype toward middle school teachers in terms of occupational personality and emotion.

Furthermore, in terms of occupational emotion about university teachers, teacher candidates had significantly lower scores in the context of positive descriptions, but significantly higher scores in the context of negative descriptions when compared with general undergraduates (Figures 1C, 2C). According to in-group favoritism, there should not have been significant differences between the scores given by teacher candidates and general undergraduates, because university teachers were an out-group for both kinds of undergraduates. The present study suggested a discrepant result that can be interpreted well by social comparison and self-evaluation maintenance (SEM) models. The SEM model is composed of reflection and comparison processes. According to social comparison theory, if another's performance is highly relevant to oneself, the comparison process will be relatively important and one will suffer by comparison with a close other's better performance. The outstanding performance of a close other could make one's own performance pale by comparison and even lead to decreased self-evaluation (Tesser et al., 1988). In fact, the SEM model suggested that persons behave in a manner that will maintain or increase self-evaluation in various ways including disparaging others (Brown, 1986; Epstein and Feist, 1988). It is common to compare middle school teachers with university teachers (Liu et al., 2007; Samek et al., 2010). As shown in Figures 1A, 2A, both teacher candidates and general undergraduates agree that university teachers have advantages in possessing more resources like salary, welfare, prospects, fame, and so on. There is no doubt that teacher candidates will become middle school teachers in the future. According to the SEM comparison process, when evaluating university teachers, teacher candidates' self-evaluations may be affected. To maintain or increase positive self-esteem, they will decrease university teachers' evaluations. Therefore, teacher candidates had more negative occupational emotions for university teachers compared with general undergraduates.

Finally, we compared the stereotypes about middle school teachers with university teachers rather with than primary teachers, which was due to the following considerations. First, undergraduates are more familiar with these two types of teachers. Specifically, now they are pursuing higher education inspired by university teachers. And they graduated from middle school not more than 4 years, but graduated from primary school at least 6 years. Therefore, the stereotypes about middle school and university teachers are clearer than the stereotypes about primary teachers (Wang, 2004; Liu et al., 2007). Second, by comparing stereotypes toward university and middle school teachers, we can understand the relative status of middle school teachers as perceived by undergraduates, because university teachers are widely considered to possess higher status and incomes in China (Wang et al., 2014). Moreover, the present study did not measure stereotypes about preschool or primary teachers because tuition-free teacher candidates will work as middle school teachers rather than as primary school or nursery teachers. It is valuable to identify the stereotypes about preschool and primary teachers held by undergraduates majoring in nursery and elementary education in the future.

In sum, the present study develops a three-factor (SCM) about middle school and university teachers, and found differences between the stereotypes held by tuition-free teacher candidates and general undergraduates. Simultaneously, we proposed that this three-factor stereotype model can be used as a potential tool to assess teacher candidates' actual willingness to work as middle school teachers, because of a significant correlation between stereotypes held by students and their willingness to work as middle school teachers. To some degree, it is good for tuition-free teacher candidates to form appropriate stereotypes about middle school teachers and form a clear career plan. Despite its contributions, the present study has certain limitations. An insufficient sample size may limit the generalizability of our findings. Future research could increase the sample size. In addition, the present model may not be applicable in other countries with different cultures. Thus, it needs further investigation in settings outside China.

## Author contributions

YGC, Substantial contributions to the design of the work, and revising the paper critically for important intellectual content. YXZ, Completing the whole study and be responsible for writing the paper. YFZ, CHP, Giving a great deal of help during the acquisition, analysis and interpretation of data. What's more, and also give the pertinent suggestions for the revise of paper. YGC, YXZ, YFZ, and CHP, Final approval of the version to be published, and got agreement to be accountable for all aspects of the work in ensuring that questions related to the accuracy or integrity of any part of the work are appropriately investigated and resolved.

## Funding

This study was supported by a grant from the Key Research Institute of Humanities and Social Science in Chongqing (16SKB008).

### Conflict of interest statement

The authors declare that the research was conducted in the absence of any commercial or financial relationships that could be construed as a potential conflict of interest.
