# Supplementary material for: A Study of Teacher Stereotypes: How Do Tuition-Free Teacher Candidates and General Undergraduates Think about Middle School and University Teachers in China?
Source: Front Psychol. 2017 Apr 19;8:576. doi: 10.3389/fpsyg.2017.00576 (PMC5395631; doi:10.3389/fpsyg.2017.00576)
Supplement: Supplementary file 1 [file DataSheet1.docx]

Table S1. Descriptive words/phrases for university teachers

| Chinese | English | frequency |
| --- | --- | --- |
| 福利好 | Better benefits | 29 |
| 科研压力大 | Stressful Researching | 29 |
| 轻松的 | Relaxed | 27 |
| 博学的 | Erudite | 27 |
| 高薪的 | Well pay | 27 |
| 杰出的 | Outstanding | 26 |
| 自信的 | Confident | 25 |
| 积极进取 | Enterprising | 25 |
| 开放的 | Open | 23 |
| 幽默的 | Facetious | 23 |
| 悠闲的 | Leisurely | 22 |
| 有名望的 | Prestigious | 21 |
| 社交广 | Wide social | 20 |
| 忙碌的 | Busy | 20 |
| 舒适的 | Comfortable | 20 |
| 省心的 | Worry free | 20 |
| 作息规律强 | Regular rest | 20 |
| 有前景 | Promising | 20 |
| 民主的 | Democratic | 20 |
| 多忧虑的 | Apprehensive | 19 |
| 友善的 | Friendly | 19 |
| 成就高 | high achievement | 18 |
| 自由的 | Free | 16 |
| 傲慢的 | Arrogant | 16 |
| 工作环境好 | Good working environment | 15 |
| 少忧虑的 | Less anxious | 15 |
| 冷淡的 | Indifferent | 14 |
| 费力的 | Arduous | 13 |
| 谦虚的 | Unobtrusive | 13 |
| 偏心的 | Partial | 12 |
| 劳心的 | Worried | 12 |
| 富有的 | rich | 11 |
| 宽松的 | Loose | 10 |
| 教学压力大 | Stressful teaching | 10 |
| 奢侈的 | Luxurious | 10 |
| 辛劳的 | Laborious | 10 |
| 高尚的 | Lofty | 10 |
| 安全的 | Safe | 10 |
| 时尚的 | fashionable | 10 |
| 能干的 | capable | 9 |
| 高雅的 | Elegant | 7 |
| 省力的 | Effortless | 7 |
| 公正的 | Just | 6 |
| 知足的 | Content | 6 |
| 热情的 | Enthusiastic | 5 |
| 自大的 | pompous | 4 |
| 平易近人 | common touch | 4 |
| 艰苦的 | Hard work | 3 |
| 贪婪的 | Greedy | 3 |
| 自私的 | selfish | 3 |
| 才思敏捷 | creativeness agile | 3 |
| 束缚的 | Restricted | 2 |
| 作息规律弱 | Irregular routine | 2 |
| 简朴的 | Frugal | 2 |
| 专制的 | Autocratic | 2 |
| 平凡的 | Mediocre | 2 |
| 狡猾的 | crafty | 2 |
| 大胆的 | bold | 2 |
| 安于现状 | Complacent | 1 |
| 创新的 | innovative | 1 |

Table S2. Descriptive words/phrases for middle school teachers

| Chinese | English | frequency |
| --- | --- | --- |
| 劳心的 | Worried | 30 |
| 教学压力大 | Stressful teaching | 28 |
| 严厉的 | Rigorous | 27 |
| 多忧虑的 | Apprehensive | 23 |
| 福利好 | Better benefits | 20 |
| 费力的 | Arduous | 20 |
| 艰苦的 | Hard work | 20 |
| 低薪的 | Poor pay | 20 |
| 忙碌的 | Busy | 20 |
| 平凡的 | Mediocre | 20 |
| 负责的 | responsible | 20 |
| 谦虚的 | Unobtrusive | 19 |
| 偏心的 | Partial | 19 |
| 简朴的 | Frugal | 18 |
| 热情的 | Enthusiastic | 18 |
| 辛劳的 | Laborious | 18 |
| 古板的 | Outdated | 17 |
| 束缚的 | Restricted | 15 |
| 知足的 | Content | 15 |
| 高尚的 | Lofty | 15 |
| 公正的 | Just | 14 |
| 才智平庸 | Mediocre intelligence | 14 |
| 积极进取 | Enterprising | 14 |
| 成就低 | Low achievement | 13 |
| 安于现状 | Complacent | 12 |
| 专制的 | Autocratic | 12 |
| 有名望的 | Prestigious | 12 |
| 工作环境好 | Good working environment | 11 |
| 安全的 | Safe | 10 |
| 社交窄 | Narrow social | 10 |
| 轻松的 | Relaxed | 10 |
| 名望低的 | Low fame | 10 |
| 博学的 | Erudite | 10 |
| 开放的 | Open | 10 |
| 幽默的 | Facetious | 10 |
| 冷淡的 | Indifferent | 10 |
| 凶悍的 | Severe | 10 |
| 友善的 | Friendly | 10 |
| 保守的 | Conservative | 10 |
| 省力的 | Effortless | 10 |
| 悠闲的 | Leisurely | 10 |
| 耐心的 | staminal | 10 |
| 前景窄 | Narrow prospect | 7 |
| 作息规律强 | Regular rest | 7 |
| 低俗的 | Vulgar | 6 |
| 社交广 | Wide social | 5 |
| 杰出的 | Outstanding | 5 |
| 舒适的 | Comfortable | 5 |
| 有前景 | Promising | 5 |
| 自由的 | Free | 4 |
| 宽松的 | Loose | 4 |
| 贪婪的 | Greedy | 4 |
| 民主的 | Democratic | 4 |
| 作息规律弱 | Irregular routine | 3 |
| 小气的 | stingy | 3 |
| 科研压力大 | Stressful Researching | 2 |
| 傲慢的 | Arrogant | 2 |
| 自信的 | Confident | 2 |
| 工作环境差 | Poor working environment | 2 |
| 自卑的 | Inferior | 2 |
| 少忧虑的 | Less anxious | 2 |
| 高薪的 | Well pay | 2 |
| 福利差 | Inadequate benefits | 2 |
| 危险的 | Dangerous | 2 |
| 势利的 | snobbish | 2 |
| 落后的 | laggard | 1 |
| 可怕的 | grisly | 1 |
