# Supplementary material for: A Study of Teacher Stereotypes: How Do Tuition-Free Teacher Candidates and General Undergraduates Think about Middle School and University Teachers in China?
Source: Front Psychol. 2017 Apr 19;8:576. doi: 10.3389/fpsyg.2017.00576 (PMC5395631; doi:10.3389/fpsyg.2017.00576)
Supplement: Supplementary file 2 [file DataSheet2.docx]

**Appendix**

All positive and negative words/phrases displayed by English and Chinese as follows.

**Positive phrases or words：**Enthusiastic，热情的；Friendly，友善的；Enterprising，积极进取；Just，公正的；Democratic，民主的；Erudite，博学的；Confident，自信的；Facetious，幽默的；Outstanding，杰出的；Unobtrusive，谦虚的；Wide social，社交广；Effortless，省力的；Leisurely，悠闲的；Free，自由的；Worry free，省心的；Less anxious，少忧虑的；Loose，宽松的；Comfortable，舒适的；Relaxed，轻松的；Well pay，高薪的；Prestigious，有名望的；Promising，有前景；Frugal，简朴的；Better benefits，福利好；Safe，安全的；Open，开放的；Elegant，高雅的；Good working environment，工作环境好；Content，知足的；high achievement，成就高；Regular rest，作息规律强；Lofty，高尚的；

**Negative phrases or words：**Arrogant，傲慢的；Autocratic，专制的；Outdated，古板的；Greedy，贪婪的；Mediocre intelligence，才智平庸；Inferior，自卑的；Indifferent，冷漠的；Severe，凶悍的；Partial，偏心的；Luxurious，奢侈的；Conservative，保守的；Narrow social，社交窄；Worried，劳心的；Arduous，费力的；Laborious，辛劳的；Stressful teaching，教学压力大；Busy，忙碌的；Apprehensive，多忧虑的；Rigorous，严厉的；Restricted，束缚的；Poor pay，低薪的；Dangerous，危险的；Mediocre，平凡的；Low achievement，成就低；Low fame，名望低；Stressful Researching，科研压力大；Hard work，艰苦的；Poor working environment，工作环境差；Inadequate benefits，福利差的；Irregular routine，作息规律弱；Narrow prospect，前景窄；Vulgar，低俗的；
